# *PTPN11* in cartilage development, adult homeostasis, and diseases

**DOI:** 10.1038/s41413-025-00425-0

**Published:** 2025-05-16

**Authors:** Wentian Yang, Véronique Lefebvre

**Affiliations:** 1 Department of Orthopaedic Surgery, Brown University Alpert Medical School and Rhode Island Hospital, Providence, RI USA; 2 Division of Orthopaedic Surgery, Department of Surgery, Children’s Hospital of Philadelphia, Philadelphia, PA USA

**Keywords:** Bone, Pathogenesis

## Abstract

The SH2 domain-containing protein tyrosine phosphatase 2 (SHP2, also known as PTP2C), encoded by *PTPN11*, is ubiquitously expressed and has context-specific effects. It promotes RAS/MAPK signaling downstream of receptor tyrosine kinases, cytokine receptors, and extracellular matrix proteins, and was shown in various lineages to modulate cell survival, proliferation, differentiation, and migration. Over the past decade, *PTPN11* inactivation in chondrocytes was found to cause metachondromatosis, a rare disorder characterized by multiple enchondromas and osteochondroma-like lesions. Moreover, SHP2 inhibition was found to mitigate osteoarthritis pathogenesis in mice, and abundant but incomplete evidence suggests that SHP2 is crucial for cartilage development and adult homeostasis, during which its expression and activity are tightly regulated transcriptionally and posttranslationally, and by varying sets of functional partners. Fully uncovering SHP2 actions and regulation in chondrocytes is thus fundamental to understanding the mechanisms underlying both rare and common cartilage diseases and to designing effective disease treatments. We here review current knowledge, highlight recent discoveries and controversies, and propose new research directions to answer remaining questions.

## Introduction

Cartilage is a distinctive connective tissue. Its unique cell type, the chondrocyte, produces an extracellular matrix (ECM) that is very abundant and primarily composed of tissue-specific types of collagens, proteoglycans, and non-collagenous proteins. The formation of cartilage primordia establishes the primary skeleton of the embryo. This complex developmental process initiates as extracellular cues lead multipotent mesenchymal stem or progenitor cells (MSPCs) to commit to the chondrocyte lineage and adopt differential differentiation programs.^1–3^ These programs allow cartilage anlagen to progressively evolve into growth plate cartilage (GPC) and articular cartilage (AC), namely in future long bones, and into elastic cartilage or fibrocartilage in other body parts, including the nose, ears and menisci. GPC is a temporary structure, entirely responsible for prenatal and postnatal lengthening of long bones.^4,5^ In contrast, AC is a permanent structure that cushions the ends of opposing bones in joints. Its main roles are to absorb impacts from mechanical loading and to reduce friction between bones during movement. Once formed, GPC and AC keep small numbers of progenitor cells and are devoid of blood vessels, lymphatics, and nerves. Therefore, their innate repair capacity is notoriously limited.^6–8^ In this regard, deciphering their biology is paramount for developing modalities to prevent, regenerate, or treat GPC and AC malformations and injuries, and aging-related AC degeneration.

Elaborate molecular networks govern cartilage development and adult homeostasis.^2,9,10^ At the beginning of chondrogenesis, multiple signaling factors, e.g., bone morphogenetic proteins (BMPs)^11^ and fibroblast growth factors (FGFs),^12^ and transcription factors (TFs), e.g., SOX9, initiate the condensation of skeletogenic mesenchymal cells at the sites of future skeletal structures.^13^ This condensed mesenchyme broadly expresses SOX9 and produces cell-cell adhesion and ECM proteins.^8,14^ While the peripheral cell layers develop into perichondrium, which maintains multipotent skeletogenic progenitors, cells in the condensation core commit to chondrogenesis. They are small and round, and start producing an abundant cartilage-specific ECM. They then proceed in a temporally and spatially staggered manner to multiple steps of growth plate-specific maturation. They first flatten and pile up in columns along the axis of longitudinal growth and actively proliferate, until they exit the cell cycle and undergo prehypertrophic differentiation, followed by hypertrophic and then terminal maturation. Each step is characterized by expression of specific markers, such as an increase in *Sox9*, *Fgfr3*, and *Runx2* expression as columnar cells reach prehypertrophy, activation of *Ihh*, *Pth1r*, and *Col10a1* at the prehypertrophic stage, and downregulation of all the above-mentioned markers, except for *Runx2* and *Col10a1* at the hypertrophic stage.^15^ Finally, terminal chondrocytes activate early osteoblast progenitor markers, such as *Sox4*, *Rankl*, and *Mmp13*. They then either die or differentiate into osteoblasts that participate in endochondral ossification, that is, remodeling of the cartilage tissue into bone trabeculae interspersed with highly vascularized bone marrow.^16–19^ Active crosstalks between perichondrial cells and chondrocytes involve many types of growth and differentiation factors that tightly regulate not only the organization and dynamic activities of the growth plate, but also the maturation of the perichondrium into periosteum and eventually cortical bone. Genetic variants that alter these crosstalks, chondrocyte activities or the ECM composition and organization cause a large family of mild to very severe skeletal dysplasias. These disorders are typically characterized by proportionate or disproportionate short stature and by generalized or localized skeleton malformations of varying degrees.

AC develops with a temporal delay relative to GPC. When embryonic cartilage primordia start to overtly give rise to GPC, presumptive joint regions remain as condensed mesenchyme. They are made of *Gdf5*^*+*^ cells, also called interzone cells, which are common progenitors of articular chondrocytes, synovial lining fibroblasts, and intra-articular ligament tenocytes.^10,20–24^ All joint structures, including the synovial cavity, start developing in fetuses, but articular chondrocytes do not overtly differentiate and form superficial, intermediate, radial, and calcified zones until a late juvenile age in mice. Mature AC maintains its cellularity and phenotype largely intact throughout life via mechanisms that are still incompletely understood. In contrast, GPC becomes thinner and poorly active beyond puberty, and while it is entirely eroded soon afterwards in humans and most mammals, resulting in growth arrest, it is maintained throughout life in rodents, allowing adult animals to keep growing slowly. Mechanical and biological stimuli are believed to be crucial to maintain adult AC homeostasis. Consequently, any insults, e.g., injury, inflammation, or genetic variants, that alter these stimuli and tissue integrity lead to AC degeneration, a main feature of such joint diseases as osteoarthritis (OA).

As in many other physiological and pathological developmental processes, posttranslational protein modifications (PTMs) are pivotal to transduce or modify biological and mechanical signaling in GPC and AC from embryogenesis onwards. They can determine either activation or deactivation of intracellular signaling cascades and transcriptional programs. PTMs include but not limited to phosphorylation, acetylation, ubiquitination, and sumoylation of specific amino acid residues. The present review focuses on protein tyrosyl phosphorylation and dephosphorylation.^25–27^ The balance between these two opposite events is maintained by the coordinated actions of protein tyrosine kinases (PTKs) and phosphatases (PTPs).^25^ To date, most of the work on PTMs in cartilage has focused on PTK-mediated protein phosphorylation.^28–30^ Limited investigations have been conducted on PTP-mediated dephosphorylation, even though their functional consequences can be equally profound. In recent years, interest in studying PTPs in cartilage biology and diseases has increased, stimulated by the vision that a detailed understanding of their functions may suggest novel medication and preventive means to build, maintain, or restore healthy GPC and AC. This review specifically focuses on the roles and regulation of the Src homology-2 (SH2) domain-containing protein tyrosine phosphatase 2 (SHP2) in cartilage development and diseases, with discussions of consensus and controversial knowledge. Important aspects of SHP2 in other biological processes have been reviewed elsewhere.^31,32^

## SHP2 gene and protein structure, function and regulation

SHP2 is encoded by *PTPN11* and forms with SHP1 (encoded by *PTPN6*) a subfamily of non-receptor PTPs.^33^ In mammals, SHP1 is mainly expressed in lympho-hematopoietic and epithelial cells, whereas SHP2 is expressed ubiquitously, although at variable levels across tissues.

**Fig. 1:**
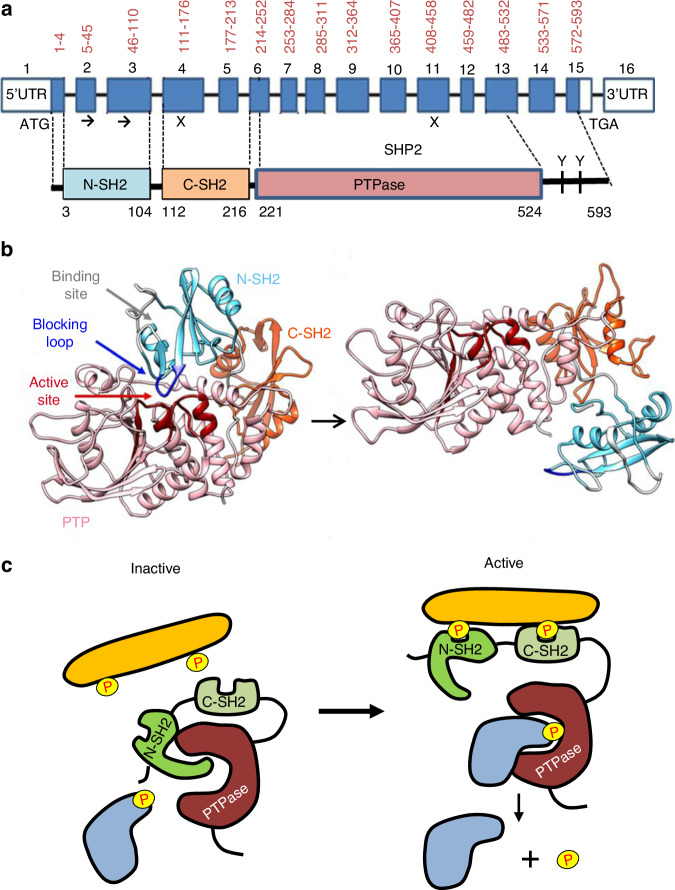
SHP2 genomic organization, protein structure and regulatory mechanisms. **a** Diagrams depicting the structure and functional N-SH2, C-SH2, and PTPase domains of SHP2 and its two tyrosyl residues Y542 and Y580 in the carboxy-terminal tail, which have signaling capability upon phosphorylation in response to certain stimuli. Note: **→**, in-frame exon; **x**, out-of-frame exon. Amino acid numbers for each exon and functional domain are marked on top and bottom, respectively. **b** Crystallographic structure demonstrating the closed and autoinhibited state (left) and the open and active state (right) of SHP2. The active site (red) of the PTP domain (pink) is blocked by the N-SH2 domain (light blue). The C-SH2 domain is colored orange. Adapted with permission from ref. ^34^ **c** Schematic model of SHP2 autoinhibition and activation. In the basal level, SHP2 is autoinhibited by the N-SH2 domain, blocking the active site. Upon binding to its signaling partners with its SH2 domains, an open and active conformation is established

Human *PTPN11* spans 91 kb at 12q24.13 and mouse *Ptpn11* spans 61 kb at 5q. In both species, the first and 15th exons contain the first and last codons, respectively, and the 16th exon is untranslated (Fig. 1a). The SHP2 protein product possesses a tandem of two SH2 domains (SH2-N and SH2-C, encoded by the exons 2–3 and 4–5, respectively), a catalytic domain (PTPase, encoded by the exons 6–13) and a C-terminal tail with tyrosine phosphorylation sites (encoded by the exons 14–15). Crystal structure resolution of SHP2 indicated that the SH2 domains bind non-covalently to the PTP domain in the basal state, leading to auto-inhibition of catalytic activity by blocking access of substrates to the active site (Fig. 1b).^34,35^ Upon exposure to extracellular stimuli, the SH2 domains bind to growth factor receptors and tyrosine-phosphorylated docking proteins (Fig. 1c). This recruitement of SHP2 to the vicinity of its substrates catalyzes dephosphorylation. Notably, mice with a deletion of the *Ptpn11* exon 3, which encodes most of the N-terminal SH2 domain and is an in-frame exon (see Table 1), display multiple defects in mesoderm patterning^36^ and die between the embryonic days 8.5 (E8.5) and E10.5, likely due to SHP2 gain of function. In contrast, *Ptpn11*-null mice, generated by deleting exon 2*, die between E3.5 and E6.5, thus around the implantation stage.^37^ These findings demonstrated that SHP2 is crucial in early embryogenesis.

**Table 1:** Mouse models with SHP2 knockin and knockout in bone and cartilage

KO or cKO	*Ptpn11* mutation	Effect on gene expression and protein	Phenotype	Refs.
Global KO	*Ex3*^*-/-*∆46-110^ (in-frame exon)	The mutant allele is expressed at 25% of WT allele with unknown cause. The protein lacks most of the SH2-N domain and has increased PTP activity.	*Ex3*^*-/-*^ mutants die between E8.5 and 10.5 with defects in gastrulation, mesoderm patterning, and FGF signaling. Heterozygotes appear normal.	^36^
Global KO	*Ex2*^*-/-*∆6-45^(in-frame exon)	Deletion of the N-terminal portion of the SH2-N domain, resulting in a truncated SHP2 protein.	*Ex2*^*-/-*∆6-45^ mutants die before E10.5 of unknown cause. Heterozygotes appear normal.	^152^
Global KO	Ex2^*-/-∆6-45^(in-frame exon)	*Ptpn11* null allele generated by replacing exon 2 with an E. coli β-galactosidase gene knock-in.	Ex2*^-/-∆6-45^ is a null allele and causes peri-implantation lethality due to elevated trophoblast cell death. Heterozygotes have normal life.	^37^
Chimeric KO	*Ex3*^*-/-*∆46-110^(in-frame exon)	*Ptpn11Ex3*^*-/-*∆46-110^*/Ptpn11*^*+/+*^ chimeras	*Ex3*^*-/-*∆46-110^ cells fail to contribute to the limb bud progress zone expansion and thus to limb outgrowth.	^36^
cKO with *Prrx1-Cre*	*Ex4*^*fx*∆111-176^ (out-of-frame exon)	Efficient inactivation of *Ptpn11* in limb bud mesenchyme and partial inactivation in sternal and cranial mesenchyme	Homozygous mutants exhibit postnatal growth retardation, limb and chest deformity, and defective mineralization of calvarial bones is associated with an absence of osteoblasts.	^107,153^
cKO with *Cd4-Cre*^*a*^	*Ex4*^*fx*∆111-176^ (out-of-frame exon)	Efficient inactivation of *Ptpn11* in CD4^+^ T cells and *Cd4-Cre+* chondrocytes.	Homozygous mutants develop kyphosis, arthritis, bony fusion, and have defects in GPC closure. Skeletal phenotypes were proposed to mimick human ankylosing spondylitis.	^104^
cKO with *Agc1-CreER*	*Ex4*^*fx*∆111-176^ (out-of-frame exon)	Efficient inactivation of *Ptpn11* in chondrocytes upon tamoxifen injection	SHP2 inactivation in *Acan*^*+*^ chondrocytes attenuates surgery-evoked PTOA by reducing the expression of uridine phosphorylase1.	^111^
cKO with *UBC-CreER*	*Ex4*^*fx*∆111-176^ (out-of-frame exon)	Ubiquitious inactivation of *Ptpn11* in cells upon tamoxifen induction.	Homozygous mutants display severe hematopoietic disorders and skeletal abnormalities, including kyphosis, scoliosis, and defective osteoclastogenesis.	^154^
cKO with *Col2a1-CreER*	*Ex4*^*fx*∆111-176^ (out-of-frame exon)	Efficient inactivation of *Ptpn11* in *Col2a1*^*+*^ mesenchymal cells and chondrocytes upon tamoxifen induction.	Homozygous mutants develop kyphoscoliosis, enchondroma, and osteochondroma-like lesions, mimicking human METCDS. Lesions display defective ERK phosphorylation but enhanced IHH signaling.	^94,108^
cKO with *LysM-Cre*	*Ex4*^*fx*∆111-176^ (out-of-frame exon)	Inactivation of *Ptpn11* in Mϕ and myeloid cells	Attenuated PTOA through reduced synovial joint inflammation.	^115^
cKO with *Prrx1-Cre*	*Ex11*^*fx*∆408-458^(out-of-frame exon)	*Ptpn11* inactivation efficient in limb bud mesenchyme and partial in sternal and cranial mesenchyme by destabilizing mRNA as a result of exon 11 deletion	Mutants have defects in endochondral and intramembranous ossification and display growth retardation and chondrodysplasia, with substantial expansion of pre- and hypertrophic chondrocytes, increased expression of SOX9-responsive genes, and long hairs growing on the forelimb and hindlimbs.	^90,91^
cKO with *Prrx1-CreER*	*Ex11*^*fx*∆408-458^(out-of-frame exon)	Inducible inactivation of *Ptpn11* efficient in limb bud mesenchyme and partial in sternal and cranial mesenchyme	Mutants develop osteochondroma-like lesions in the metaphysis of tubular bones and vertebrae.	^91^
cKO with Prg4-CreER	*Ex11*^*fx*∆408-458^(out-of-frame exon)	Inducible inactivation of *Ptpn11* in *Prg4*^*+*^ AC cells as a result of E11 deletion	Increased AC cellularity and thickness and anabolic gene expression.	^93^
cKO with *Col2a1-Cre*	*Ex11*^*fx*∆408-458^(out-of-frame exon)	Inactivation of *Ptpn11* in *Col2a1*^*+*^ chondrocytes	Mutants degenerate around E11.5 with unknown causes.	^16^
cKO with *Col2a1-CreER*	*Ex11*^*fx*∆408-458^(out-of-frame exon)	Inducible inactivation of *Ptpn11* in *Col2a1*^*+*^ chondrocytes	Juvenile mutants have elongated GPC and aging mutants develop METCDS -like lesions, featuring kyphoscoliosis and formation of enchondroma and osteochondroma around the epiphysis of tubular bones and vertebrae due to elevated cell proliferation and delayed terminal differentiation.	^16,92^
cKO with *Acan*^*CreER*^	*Ex11*^*fx*∆408-458^(out-of-frame exon)	Inducible inactivation of *Ptpn11* in *Acan*^*+*^ chondrocytes	Juvenile mutants have elongated GPC,increased body length, and increased AC thickness and cellularity. They develop METCDS -like disease with joint and vertebral deformation when aging. GPC remains active during adulthood.	^93^
cKO with *CD4-Cre*	*Ex11*^*fx*∆408-45^ (out-of-frame exon)	Efficient inactivation of *Ptpn11* in CD4^+^ T cells and *Cd4-Cre*^*+*^ chondrocytes	T cell development is normal, but the mutants grow METCDS -like cartilage tumors due to SHP2 deletion in *Cd4-Cre*^*+*^ chondroid cells in epiphyseal cartilage.	^95,96^
cKO with *Col10a1-Cre*	*Ex11*^*fx*∆408-458^(out-of-frame exon)	Efficient inactivation of *Ptpn11* in *Col10a1*^*+*^ hypertrophic chondrocytes	Defective osteogenic conversion of GPC hypertrophic chondrocytes due to elevated SOX9 abundance.	^16^
cKO with *Sp7-Cre*	*Ex11*^*fx*∆408-458^(out-of-frame exon)	Efficient inactivation of *Ptpn11* in *Sp7*^*+*^ stromal, gut crypt stem cells and osteoblastic cells	Juvenile lethality, expanded GPC, defective ossification, and bone marrow vasculopathy.	L.W & W.Y (submitted)
cKO with *Sp7-CreER*	*Ex11*^*fx*∆408-458^ (out-of-frame exon)	Inducible inactivation of *Ptpn11* in *Sp7*^*+*^ stromal and osteoblastic cells	Postnatal administration of tamoxifen impairs endochondral ossification.	L.W & W.Y (submitted)
cKO with *Bglap-Cre*	*Ex11*^*fx*∆408-458^ (out-of-frame exon)	Inducible inactivation of *Ptpn11* in osteocalcin^+^ osteoblasts	Mutants have defective endochondral ossification and develop METCDS -like lesions when aging due to aberrant proliferation of GPC chondrocytes.	^137^
cKO with *LysM*^*Cre*^	*Ex11*^*fx*∆408-458^ (out-of-frame exon)	Inactivation of *Ptpn11* in macrophages and myeloid cells	Mutants have relatively normal myelopoiesis but develop osteopetrosis when aging due to compromised osteoclast formation.	^116,155^
cKO with *Ctsk*^*Cre*^	*Ex11*^*fx*∆408-458^ (out-of-frame exon)	Inactivation of *Ptpn11* in *Ctsk*^*+*^ osteoclasts and perichondral and periosteal osteochondro-progenitors.	Mutants grow many osteochondromas and enchondromas, mimicking human METCDS as a result of SHP2 deletion in the *Ctsk*^*+*^ chondroprogenitors in the groove of Ranvier and perichondrum. These mice also have severe osteopetrosis due to defective osteoclastogenesis.	^90,155^
cKO with *Fsp1-Cre*	*Ex11*^*fx*∆408-458^	*Fsp1-Cre* targets fibroblasts that expess fibroblast specific protein-1 (S100A4) during development after embryonic day 8.5.	Osteochondroma-like lesions on bone surface or at bone-ligament insertion sites.	^92,156^
cKO with *Wnt1-Cre*	*Ptpn11 ∆Ex(3,4)*^*fx*^ (out-of-frame exon)	*Wnt1-Cre* is active in premigratory neural crest cells (NCCs) residing in the dorsal neural tube, and is extinguished as NCCs migrate away from the neural tube.	Mutant NCCs have normal migratory and proliferative patterns, but fail to migrate into the developing outflow tract. Embryos display persistent truncus arteriosus, abnormalities of the great vessels, and severe craniofacial defects.	^157^
KI	*Ptpn11*^*D61G*^	Ex3^D61G^, Noonan syndrome– associated *PTPN11* mutation.	D61G homozygotes die in utero. Heterozygotes have reduced postnatal viability, short stature, craniofacial abnormalities, and myeloproliferative disease similar to Noonan syndrome.	^158^
KI	*Ptpn11*^*Y279C*^	Ex7^Y279C^, one of the most common LEOPARD alleles.	Y279C/+ mice recapitulate human LS clinical feafures, manifesting short stature, craniofacial dysmorphism, morphologic, histologic, echocardio-graphic features, and molecular evidence of hypertrophic cardio-myopathy (HCM).	^74^
cKI	*Ptpn11 Q79R*	Using *Wnt1-Cre* to express SHP2 Q79R in neural crest cells.	Neural crest expression of SHP2Q79R results in craniofacial defects and growth retardation of a subset of the bony and cartilaginous structures of the skull.	^159^

^a^The original description about this Cre mouse line cannot be identified. So, whether it harbors a Cre transgene or knock-in allele coul not be determined

SHP2 itself has been reported to be regulated by posttranslational modifications (PTM), in particular, by phosphorylation of Y63, Y279, Y542, and Y580 upon growth factor stimulation.^38–42^ ABL (Abelson murine leukemia viral oncogene homolog) kinases can phosphorylate Y279, Y542, and Y580,^40,42^ and PDGFRβ too can phosphorylate Y542.^42,43^ Whether phosphorylation of Y580 affects SHP2 activity and function remains controversial. Conflicting data have been presented regarding adapter or enzymatic roles for Y542/580-phosphorylated SHP2 in the RAS/ERK pathway activation.^43–46^ On the contrary, phosphorylation of Y63 and Y279, which reside in the SH2-N/PTP domain interface and PTP domain, respectively, has been reported to alter the signaling ability of SHP2 and to affect cell proliferation.^40^ Further studies are required to elucidate the exact mechanisms by which phosphorylation at these residues regulates RAS/ERK activation and mitogen-mediated proliferation. It has been also reported that SHP2 S576 and S591 can be phosphorylated by PKC isoforms, but the significance of these modifications is unknown.^47^

Despite intense research in the past decades, the actions of SHP2 remain incompletely understood. Genetic and biochemical evidence demonstrates that SHP2 is required for RAS/ERK pathway activation by most, if not all, RTKs, as well as by G protein-coupled receptors (GPCRs), cytokine receptors, and integrins. However, some data also imply that SHP2 may function downstream of or in parallel to RAS.^48^ SHP2 has been reported to interact with diverse signaling molecules, such as GABs (growth factor receptor-bound protein 2 (GRB2)-associated binding proteins), FRS (fibroblast growth factor receptor substrate proteins), IRS1/2 (insulin receptor substrate 1/2 proteins), p85 (phosphatidylinositol 3-kinase [PI3K], regulatory subunit), STAT1/3/5 (signal transducer and activator of transcription 1/3/5), and SPROUTY proteins (RTK signaling antagonists).^49^ SPROUTY proteins are a family of cytoplasmic proteins that regulate signaling mediated by RTKs, particularly the activation of the MAPK pathway. The role of SHP2 in relation to SPROUTY protein varies depending on the cellular context. In some cases, activation of SHP2 enhances SPROUTY activity, reinforcing its function as a feedback inhibitor of RTK signaling. Conversely, in other circumstances, SHP2 can reduce SPROUTY activity by dephosphorylating inhibitory phosphotyrosine residues, which allows for more robust signal transduction.^50,51^ Moreover, SHP2 is essential for the recruitment of SOS, a guanine nucleotide exchange factor that facilitates RAS activation and initiates the Raf/MEK/ERK signaling cascade.^52^ On the other hand, RasGAP negatively regulates RAS activity by promoting the hydrolysis of GTP to GDP, thus converting RAS from its active form (Ras-GTP) to its inactive form (Ras-GDP).^53^ SHP2 modulates RasGAP activity by dephosphorylating specific tyrosine residues in the RasGAP complex or its associated proteins.^54^ Through this modulation, SHP2 helps ensure that RAS activation is properly regulated, thereby maintaining cellular homeostasis. Accordingly, in addition to its role in the RAS/ERK pathway activation, SHP2 also differentially regulates the PI3K pathway downstream of RTKs, reportedly suppressing S6K1 (ribosomal protein s6 kinase 1) activation by stimulating AMPK (5’ adenosine monophosphate (AMP)-activated protein kinase) and JNK (mitogen-activated c-JUN N-terminal kinase), NF-κB (nuclear factor kappa-B), RHO (RAS homology family member), and NFAT (nuclear factor of activated T cells) activation in various settings.^55,56^ Moreover, SHP2 has been reported to regulate mitochondrial homeostasis through dephosphorylating ANT1 at Tyr-191^57,58^ and cell autophagy by modulating AKT or mTOR signaling,^59^ suggesting that SHP2 carries out various functions by targeting substrates in a cellular context-specific manner.

## SHP2 variants in human diseases with cartilage manifestation

*PTPN11* is ubiquitously expressed and variants causing either loss or gain of SHP2 function have been associated with a spectrum of human diseases. These include chronic and acute myelomonocytic leukemia (CML, AML),^60,61^ hypertrophic cardiomyopathy (HCM),^62^ Noonan and LEOPARD^63,64^ syndromes, and metachondromatosis.^65^ Regarding the involvement of SHP2 variants in hematopoietic and other organ/tissue disorders, multiple reviews are available.^31,66^ Here we focus on SHP2-related diseases that have skeletal manifestations.

### Noonan and LEOPARD syndromes

Noonan syndrome (NS1, MIM: 163950) is an autosomal dominant disorder characterized by unusual facial features, webbed neck, proportionate short stature, cardiac abnormalities, and variably penetrant chest and spine deformities.^67^ *PTPN11* variants account for about 40% of NS1 cases and for about 90% of cases with LEOPARD syndrome (LPRD1, MIM: 151100), another rare multiple congenital anomalies condition, featuring Lentigines, ECG conduction abnormalities, Ocular hypertelorism, Pulmonic stenosis, Abnormal genitalia, Retardation of growth, and Deafness.^68^ Most of the somatic variants in NS1 are clustered in the PTPase and N-SH2 domains, altering the autoinhibition mechanism, and thereby resulting in SHP2 gain of function (GOF).^69,70^ In contrast, all LPRD1 variants encode catalytically defective, but not inactive, forms of SHP2, leading to impaired activation of the RAS/ERK signaling pathway.^71^ Although NS1 and LRPD1 variants have opposite effects on SHP2 activity, NS1 and LRPD1 patients share multiple overlapping clinical traits, including short stature and facial dysmorphism.^72^ Consistent with these findings, Tajan et al. reported that GPC length was reduced in *Ptpn11*^*D61G/+*^ mice, which model NS. This was primarily due to a shorter hypertrophic zone, which was correlated with RAS/ERK hyperactivation, decreased expression of chondrocyte markers (e.g., *Sox9*, *Col2a1, Acan*, and *Ihh*), and decreased alkaline phosphatase activity in mouse chondrocytes in vitro.^73^ Importantly, RAS/ERK inhibition by U0126 alleviated GPC abnormalities.^73^ However, the impact of *LPRD1* orthologous mutations on GPC was not fully characterized in the affected mutants.^74,75^ Of note, most patients with NS and LRPD1 also show laxity of small joints and sternal and elbow anomalies.^76^ Collectively, accumulated lines of evidence indicate that aberrant signaling, due to germline variants and/or altered protein functions along the RAS/MAPK pathway, are responsible for a spectrum of related genetic disorders called RASopathies, including but not limited to NS1 and LRPD1, that have distinctive facial and skeletal abnormalities in addition to cardiopathies, mental retardation, and tumor predisposition.^77,78^

### Metachondromatosis

**Fig. 2:**
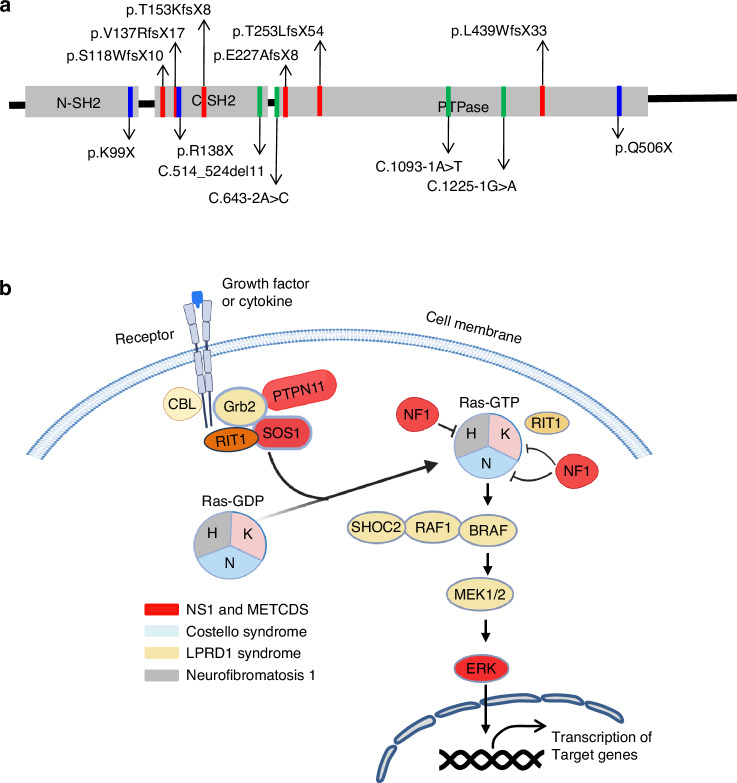
Mutations in SHP2 and its signaling partners associated with human METCDS and RASopathies. **a** Diagrams depicting METCDS variants identified in the functional domains of SHP2. Additional information can be found at https://www.uniprot.org/uniprotkb/Q06124/entry#disease_variants. **b** Schematic model of the RAS pathway, with protein components colored according to the METCDS and RASopathies caused by mutations in their genes

Metachondromatosis (METCDS, MIM: 156250) is a rare autosomal-dominant skeletal disorder, characterized by enchondromas and osteochondromas primarily in the iliac crest and tubular bone metaphyses.^79–81^ These lesions are usually asymptomatic and either regress spontaneously or transform into chondrosarcomas.^82^ *PTPN11* variants that cause METCDS represent a new category of *PTPN11* loss-of-function (LOF) variants. These variants include deletions and nonsense or splice site changes. METCDS lesions often feature loss of *PTPN11* variant heterozygosity (LOH).^65,83^ In contrast to *PTPN11* GOF variants associated with other human neoplasms,^66,84–86^ LOH of *PTPN11* variants drives cartilage tumor development, suggesting that *PTPN11* functions as a tumor suppressor in cartilage. Similar actions were also described in liver cancer.^86^ Known human METCDS variants are depicted in Fig. 2a and described in the literature.^65,87^ Whether *PTPN11* LOF variants exist in other cartilage neoplasms (e.g., chondrosarcomas) remains unclear.

The cells of origin in METCDS remain controversial.^88^ The appearance of METCDS lesions adjacent to the GP suggests that these cells reside in the GP, periosteum, perichondrium, or groove of Ranvier.^89^ In an attempt to study SHP2 function in osteoclast development in vivo, we used *Ctsk* (cathepsin K)*-Cre* to inactivate *Ptpn11* in mice. Unexpectedly, a large number of enchondromas and osteochondromas occurred in *Ctsk*^*Cre*^*;Ptpn11*^*Ex11fx/fx*^ mutants, and they exhibited the clinical features of human METCDS. Later, it was found that these neoplasms were arising from a small population of *Ctsk*^***+***^ mesenchymal stem/progenitor cells (MSPCs) located in the perichondrium, and most abundantly in the groove of Ranvier, a perichondrium region adjacent to the top of the growth plate that provides the growth plate with newly formed chondrocytes.^90^ In subsequent analogous studies using other Cre drivers, *Ptpn11* homozygous but not heterozygous inactivation in *Prrx1*^***+***^ skeletogenic progenitors,^91^ *Fsp1*^*+*^ fibroblasts (including perichondrium and periosteum cells)^92^ or *Acan*^*+*^,^93^ *Col2a1*^***+***^^16,94^ or *Cd4-Cre*^***+***^^95,96^ chondrocytes, always gave rise to METCDS-like lesions, strongly suggesting that the human disease originates from periosteal and perichondrial progenitors or chondrocytes, and that *Ptpn11* LOH in these cells causes tumorigenesis.^91^ Multiple signaling pathways regulate chondrogenesis and cartilage homeostasis.^3,4,97–100^ Since SHP2 plays a crucial role in RTK-evoked RAS/ERK pathway activation^31,101^ and since dysregulation of the RAS-MAPK signaling as a result of mutations in various genes (*B-RAF,CBL, RAS* (N, H, K), *MAP2K1, MAP2K1, PTPN11, RAF1, RIT1, SHOC2, SOS1* and *SOS2*), SHP2 is associated with the group of rare congenital diseases named RASopathies. These diseases include the previously described NS1 and LPRD1 as well as cardiofaciocutaneous syndrome, Costello syndrome, Neurofibromatosis-Noonan syndrome, and Legius syndrome. They are characterized by craniofacial anomalies, heart defects, short stature, and variable neurodevelopmental disorders^102^ (Fig. 2b). SHP2 was postulated to downregulate Indian hedgehog (*IHH*) expression through increasing ERK activation and thereby to maintain GPC chondrocytes in an immature, proliferative stage. Accordingly, cartilage tumors in *Ctsk*^*Cre*^*;Ptpn11*^*Ex11fx/fx*^, *Col2a1-CreER;Ptpn11*^*Ex11fx/fx*^ and *Ctsk*^*Cre*^*;Ptpn11*^*E4fx/fx*^ mice showed decreased ERK activation^90,94,103^ and increased *Fgf2*,^94^ *Ihh*,^90,94^ and *Pthrp*^90^ expression. Moreover, the METCDS features in *Ctsk*^*Cre*^*;Ptpn11*^*Ex11fx/fx*^ mice could be ameliorated by treatment with PF-04449913 (SMOi), an inhibitor of the IHH receptor smoothened.^90^ Similar findings were reported in an analogous study where *Cd4-Cre;Ptpn11*^*Ex4fx/fx*^ mice were treated with the SMOi Sonidegib.^104^ Thus, SHP2 activation apparently suppresses the proliferation of chondroprogenitors via ERK activation, and the loss of SHP2 lifts this suppression and results in tumorigenesis. These findings clearly show that *PTPN11* has cellular context-dependent pro- and anti-oncogenic actions, albeit via the common theme of affecting RAS/ERK pathway activation.

## Roles of SHP2 and its signaling partners in cartilage development

### Precartilaginous condensation and chondrocyte lineage specification

Homozygous deletion of *Ptpn11* is embryonically lethal in mice.^36,37^ To study the functions of SHP2 at the cellular level, Saxton et al. generated mouse chimeras derived from a mixture of wild-type and *Ptpn11*^*Ex3-/-*^ embryonic stem (ES) cells. This was the first study that uncovered an obligatory role of SHP2 in limb development. Unlike control counterparts, mutant cells failed to contribute to the embryonic limb bud progress zone, a lateral plate mesoderm-derived mesenchymal compartment that progressively gives rise to all limb skeletal structures. The *Ptpn11* alteration had minimal impact on the ability of wild-type ES cell-derived progress zone cells to proliferate. However, it significantly impaired the cells’ adhesive properties and ability to induce and maintain the ectoderm-derived apical ectodermal ridge, which plays a key role in regulating limb bud growth and proximodistal patterning.^105^ This suggested that SHP2 regulates the expression of genes essential for proper development of the early limb primordium. However, the interpretation of these initial studies was complicated by the use of *Ptpn11*^*Ex3-/-*^ cells. The *Ptpn11*^*Ex3-*^ variant indeed encode a protein that is truncated in the N-terminus and has increased PTP activity and that could thus have hypomorphic (reduced), hypermorphic (enhanced), and/or neomorphic (new) functions.

**Fig. 3:**
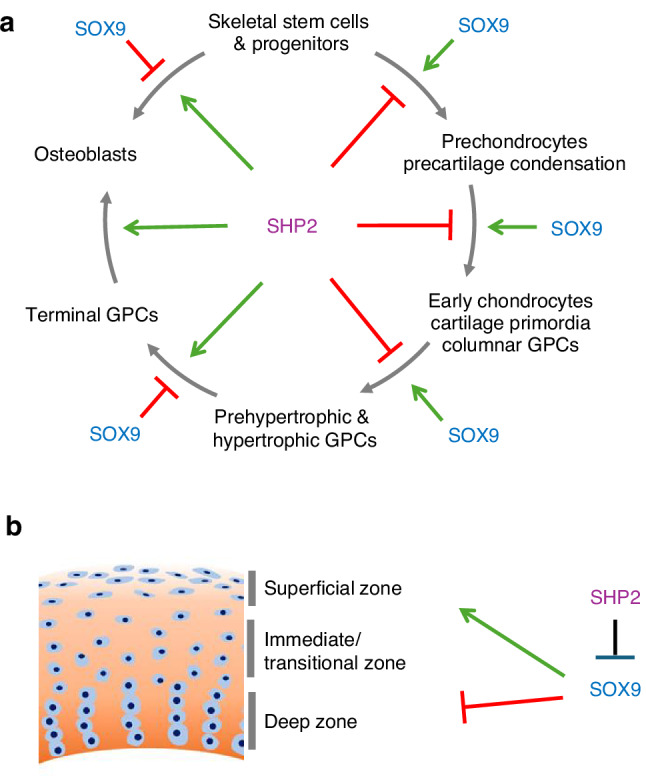
Diagram illustrating the involvement of SOX9 (blue) and SHP2 (Brown) in **a** the stage-specific regulation of chondroid cell specification, differentiation, maturation and osteoblastic conversion and **b** in articular cartilage zone maintenance. (→: promotion; --I: inhibition; GPC growth plate cartilage)

*Prrx1-Cre*, a Cre transgene driven by the paired-related homeobox gene-1 (*Prrx1*) promoter, is specifically expressed in the mouse in early limb bud mesenchyme and in a subset of sternal and cranial mesenchymal cells.^106^ Lapinski et al.^107^ reported that mice harboring *Prrx1-Cre* and *Ptpn11* conditional null alleles (*Ptpn11*^*Ex4fx/fx*^) exhibited growth retardation, limb and chest deformities, and defective calvarial ossification. At the cellular level, a massive accumulation of hypertrophic chondrocytes and an absence of osteoblasts were noted. At the molecular level, defective ERK and AKT (Ak strain transforming, also known as protein kinase B) activation were detected. In a parallel study, Zuo et al.^91^ showed similar skeletal manifestations upon ablation of SHP2 expression in the *Prrx1*^+^ cells (*Prrx1-Cre;Ptpn11*^*Ex11fx/fx*^ mice). Transcriptome analyses demonstrated that SHP2 deficiency suppressed the osteogenic program in limb bud mesenchyme, but enhanced the chondrogenic program, including the expression of the targets of the master chondrogenic transcription factor SOX9.^91^ Lineage tracing studies revealed that limb bud patterning was normal in SHP2 mutants at E10, and that cell proliferation was dramatically decreased by E11.5 (W.Y, unpublished data). Further mechanistic studies showed that SHP2 destabilized SOX9 by promoting its phosphorylation and SUMOylation at specific serine and lysine residues.^91^ Elevated SOX9 level in SHP2-deficient limb skeletogenic progenitors and their progeny is thus likely a major contributor of the enhanced chondrogenic program (Fig. 3).

### Chondrocyte maturation and osteogenic conversion

To understand how SHP2 regulates chondrocyte differentiation and maturation, we and others ablated *Ptpn11* expression from the immature chondrocyte stage using *Col2a1-Cre* and *Col2a1-CreER*,^16,92,94,108^ and in hypertrophic chondrocytes using *Col10a1-Cre*.^16,109^ Mice lacking SHP2 in the *Col2a1* lineage died during mid-gestation of unknown cause.^16^ Tamoxifen-induced SHP2 ablation at the postnatal week 2 in *Col2a1*^*+*^ cells led to elongated GPC, with a significant increase in the numbers of both proliferating and hypertrophic chondrocyte layers, to scoliosis and kyphosis by 6 weeks of age, and to osteochondroma development at a later age.^16,94^ SHP2 ablation was found to inhibit ERK1/2 activation and delay chondrocyte maturation from the early to the late hypertrophic stage.^92^ In contrast, mice with SHP2 ablation in the *Col10a1*^*+*^ cells appeared normal, but for osteopenia. Detailed analysis found that SHP2 ablation increased SOX9 protein abundance in hypertrophic chondrocytes, and halted their apoptosis or conversion into osteoblasts^16^ (Fig. 3). *Sox9* haploinsufficiency in *Col10a1-Cre;Ptpn11*^*Ex11fx/fx*^ mice restored the expression of osteogenic genes, e.g., *Ctnnb1* (beta-catenin), *Ibsp* (integrin-binding sialoprotein), and *Mmp13* (matrix metalloproteinase 13) and rescued osteogenesis,^16^ corroborating that SOX9 has a key function as a gatekeeper of chondrogenic versus osteogenic differentiation.^110^

Conceivably, some discrepancies were noticed regarding the trajectory of METCDS lesions in mice lacking SHP2 in *Col2a1*^***+***^ cells. Kim et al.^94^ did not find any lesions in *Col2a1-CreER;Ptpn11*^*Ex4fx/fx*^ mice when SHP2 inactivation was induced in adulthood (8 weeks). However, Wang et al.^16^ observed osteochondromas in *Col2a1-CreER;Ptpn11*^*Ex11fx/fx*^ mice and both osteochondromas and enchondromas in *Agc1*^*CreER*^*;Ptpn11*^*Ex11fx/fx*^ mice when SHP2 inactivation was induced in adulthood (8 weeks).^93^ Inconsistency between the results of these studies may be attributed to variations in tamoxifen administration regimens or to differences between the residual SHP2 proteins resulting from Ex4 or Ex11 deletions. Another possible explanation is the virtual absence of *Col2a1-CreER* expression versus strong expression of *Agc1*^*CreER*^ in adulthood.

## Roles of SHP2 in osteoarthritis pathogenesis

Osteoarthritis is a debilitating joint disease that is most prevalent in the elderly, but that can also occur in prime adulthood, generally as a consequence of mechanical trauma to a joint. It affects all joint tissues, but is hallmarked by the progressive loss of AC, whose healthy homeostasis is regulated by various biologic and mechanical stimuli that concertedly activate intracellular signaling cascades and TFs. Recent proceedings in understanding OA pathogenesis suggest a crucial role of SHP2 in modulating these signaling cascades. However, findings from different studies bring confusions and controversies regarding the underlying cellular and molecular mechanisms.

### Do SHP2 abundance and activity change in OA cartilage?

**Fig. 4:**
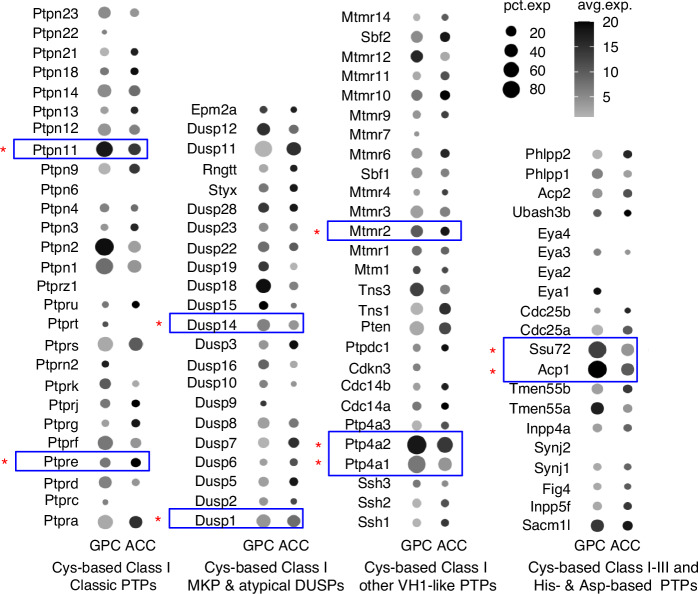
Bubble plots illustrating the expression levels of *Ptpn11* and other classic PTPs and MKPs, atypical DUSPs, and His and Asp-based PTPs in growth plate (GPC) and articular chondrocytes (ACC). Data were obtained by scRNA-seq of knee and adjacent growth plate cells of 13-day-old mice.^110^ Interestingly, all nine highly expressed PTPs found in human OA cartilage by Liu et al.^111^ (blue boxes and red stars) are highly expressed in juvenile murine ACC and GPC

By analyzing available scRNA-seq datasets, Liu et al.^111^ reported that 9 of 107 analyzed PTP-encoding genes were highly expressed in human OA AC, and that *PTPN11* topped the list. Their immunostaining and qRT-PCR analyses, however, revealed comparable levels of SHP2 protein and *PTPN11* RNA in healthy and OA AC, and in primary AC chondrocytes treated with and without IL-1β. Interestingly, by conducting PTP activity assays using DiFMUP as a substrate, they found that SHP2’s enzymatic activity was significantly increased in human OA cartilage, and they obtained a similar result through immunostaining for SHP2 Y542/Y580 phosphorylation in murine AC chondrocytes in vivo and upon IL-1β treatment in vitro.^111^ The concurrence of OA, elevated SHP2 phosphorylation (Y542/Y580), and SHP2 enzymatic activity inspired the postulation that increased SHP2 enzymatic activity may be involved in OA pathogenesis. These findings, however, were challenged by the work of Tao et al.^112^ who reported a significant increase in the abundance of SHP2 protein and *Ptpn11* transcript*s* in murine OA AC and IL-1β-stimulated mouse chondrocytes. The discrepancies between these studies raised questions on whether OA changes SHP2 activity, expression, or both, and on whether the alterations contribute to the disease progression. The answers to these questions could be instrumental to suggest new pharmacological strategies to sustain AC homeostasis and combat OA. In reviewing the studies by Liu et al.^111^ we found no convincing evidence of an increase in the activity of SHP2 itself in OA cartilage. The authors indeed carried out PTP activity assays using total cell lysates (TCL), and attributed all PTP activity measured following PHPS1 inhibition to SHP2. Conceivably, this assay may have hit other PTPs in addition to SHP2. Moreover, since earlier studies showed that only a fraction of the total SHP2 undergoes phosphorylation at Y542/Y580 in response to stimuli, the stoichiometry of SHP2 Y542/Y580 phosphorylation determined in vivo by immunostaining and in vitro by western blotting in Liu’s studies appears to be too high to fit this model.^111^ Also, whether SHP2 Y542/Y580 phosphorylation in chondrocytes reflects altered enzymatic activity or adapter function, as described in other cell types,^113,114^ remains uncertain. In an effort to resolve these confusions, we carried out analogous studies using primary murine *Acan*^*+*^ and *Prg4*^*+*^ chondrocytes and found that treatment with IL-1β impacted neither SHP2 expression nor enzymatic activity.^93^ Upon mining a murine scRNA-seq dataset,^110^ we unveiled a list of PTP genes that are highly and differentially expressed in AC and GPC, including those for SHP2, other Cys-based Class I PTPs (27), Class I mitogen-activated protein kinase phosphatases (MKPs) and atypical dual-specificity phosphatases (DUSPs, 22), and His- and Asp-based PTPs (47) (Fig. 4). Of note, the function of many of these PTPs in cartilage remains unknown. Importantly, nine of the highly expressed PTPs found in human OA cartilage by Liu et al.^111^ were robustly expressed in healthy murine AC and GPC, indicating that these PTPs are ordinarily rich in AC and GPC. Thus, whether their expression or functional activation is associated with OA pathology remains elusive. Interestingly, Sun et al.^115^ recently reported synovial accumulation of SHP2-expressing F4/80 macrophages in OA joints in both humans and mice, and showed that SHP2 ablation in these cells mitigated the severity of post-traumatic OA pathology evoked by surgical destabilization of the medial meniscus (DMM). There are several concerns about this study. First, it is unclear why SHP2 was expressed in less than 20% of synovial cells, given that the ubiquitous expression of SHP2 is well known. Second, the identity of the SHP2^**+**^ cell types was not disclosed. Third, F4/80 is a cell surface glycoprotein and SHP2 is primarily cytoplasmic, but the immunostaining and colocalization data presented in this study did not convincingly reflect these features. Fourth, it is well established that synovial inflammation has minimal impact on DMM-evoked OA, and that SHP2 ablation has a negligible effect on macrophage differentiation.^116^ Finally, the authors previously showed that ablation of SHP2 in *Acan*^+^ lineage cells greatly attenuated DMM-evoked OA, suggesting that SHP2 deletion in chondroid cells^111^ rather than in macrophages mitigated OA pathology. Conceivably, discrepancies remain regarding the roles of SHP2 in OA. Additional studies are thus warranted to address them.

### Does the SHP2/DOK1/UPP1 signaling axis play a role in OA pathogenesis?

DOK1 (downstream-of-tyrosine-kinase protein 1) belongs to the family of IRS (insulin receptor substrate) adapter proteins and is one of seven DOK family members known to function downstream of various tyrosine kinases.^117,118^ DOK family members have plekstrin homology (PH) and phosphotyrosine-binding (PTB) domains at the NH2-terminus, and SH2 target motifs (YXXP) in the C-terminus.^117^ DOK1 is preferentially expressed in immune cells and has multiple tyrosine residues that undergo phosphorylation and relay cellular signaling upon PTK activation. Phosphorylation of DOK1 at Y362 and Y398 is essential for Ras GTPase-activating protein (RasGAP) binding, for Ras and AKT activation,^119^ and for DOK1 Y449 to recruit C-terminal Src kinase (CSK) and active Src.^118,120,121^ PTPs responsible for DOK dephosphorylation remain elusive, except that SHP1 was found to dephosphorylate p62DOK in macrophages.^122^ Since DOK proteins have no enzymatic activity, their biological actions are most likely related to those of their binding partners. SHP2 was reported to bind to DOK1 in response to insulin-like growth factor 1 (IGF1) in vascular smooth muscle cells^123^ and to the epidermal (EGF) and hepatocyte (HGF) growth factors in HEK-293 cells,^124^ and DOK1 overexpressed through transfection was shown to be hyperphosphorylated at Y398 upon EGF and HGF stimulation. Using substrate-trapping mutants coupled with a high-throughput quantitative proteomics approach, Zhu et al. proposed DOK1 as a substrate of SHP2 in EGF-stimulated HEK-293 cells.^124^ Whether DOK1 is an SHP2 substrate in chondrocytes in vivo remains elusive.

**Fig. 5:**
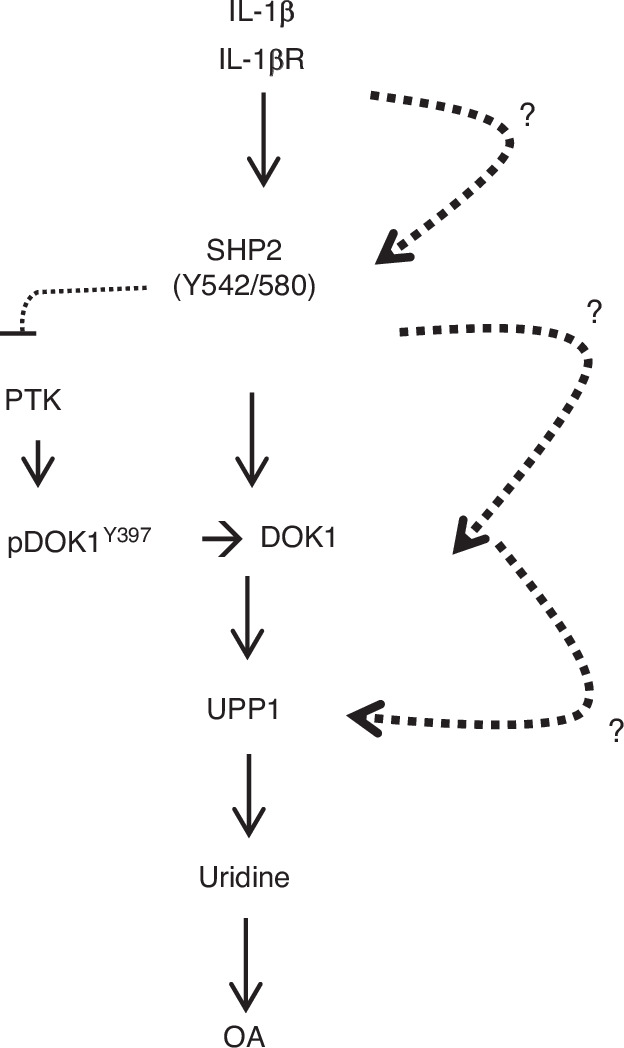
Diagram illustrating the signaling molecules and pathways evoked by IL-1β in driving OA pathogenesis described by Liu, et al.^111^ Dash lines and question marks between signaling molecues indicationg the lack of data supporting their connection. PTK protein tyrosine kinase

Liu et al. ^111^ recently described the involvement of the SHP2/DOK1/UPP1 signaling axis in OA pathogenesis (Fig. 5). Using a quantitative phosphoproteomic approach, they found DOK1 to undergo phosphorylation at Y397 in ATDC5 chondrogenic cells upon IL-1β stimulation. DOK1 phosphorylation at Y397 was augmented by SHP099 treatment. Interestingly, DOK1 was shown to interact with SHP2 upon IL-1β induction, and this interaction was impaired by SHP099 treatment in their study. The authors then concluded that DOK1 is a substrate of SHP2 in ATDC5 cells and presumably in chondroid cells in vivo. This molecular model, however, is ambiguous since it is unclear how IL-1β mediated the binding of SHP2 to DOK1. If SHP2 and DOK1 interacted via their respective SH2 domains and SH2 domain-targeting motifs “YXXP”, as reported,^123^ the “YXXP” motifs of DOK1 should be phosphorylated in response to IL-1β. There was no evidence illuminating how SHP2-DOK1 interaction was interrupted by SHP099. Moreover, no information was provided regarding the molecular link between IL-1β-evoked serine/threonine kinase activation and DOK1 Y397 phosphorylation. Therefore, the proposed signaling model seems premature. It is possible that increased DOK1 Y397 phosphorylation upon SHP099 treatment resulted from the activation of another PTK(s) rather than from impaired Y397 dephosphorylation upon SHP2 inhibition.

Uridine phosphorylase 1 (UPP1) catalyzes the reversible phosphorolytic cleavage of uridine and deoxyuridine, and DOK1 acts as an adapter protein, but the molecular link proposed in Liu’s work^111^ between DOK1 and UPP1 in response to IL-1β signaling is unclear and lacks the support of direct evidence. In a follow-up study, we examined DOK1 Y397 phosphorylation and UPP1 protein and gene expression in *Prg4*^*+*^ and *Acan*^*+*^ articular chondrocytes. In western blot analysis of cell lysates, neither SHP2 deletion nor IL-1β induction was found to impact DOK1 Y397 phosphorylation. IL-1β upregulated UPP1 protein and RNA levels, but these responses were independent of SHP2.^93^ In sum, we did not find evidence supporting the SHP2/DOK1/UPP1 signaling axis in murine chondrocytes. Whether these discrepancies reflect differences in the cell types used and/or altered UPP1 expression in OA tissues awaits future investigation.

### Is β-catenin a target of SHP2 in cartilage?

**Fig. 6:**
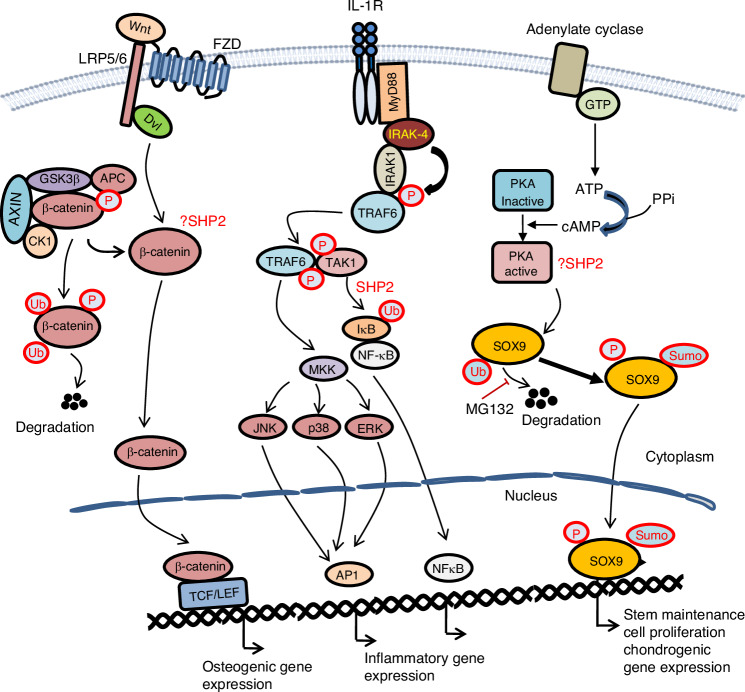
Diagrams depicting mechanism(s) by which SHP2 may regulate SOX9 and β-catenin protein stability through modifying its phosphorylation, SUMOylation, and ubiquitin-mediated proteasomal degradation in response to Wnt, IL-1β, and cAMP-evoked signlaing pathway activation in chondrocytes. MG132: proteosome inhibitor

In contrast to the work by Liu et al.^111^ Tao and colleagues found a significant increase in the abundance of SHP2 protein and *Ptpn11* transcripts in murine chondrocytes upon IL-1β induction.^112^ They also reported that SHP2 overexpression exacerbated IL-1β-evoked cartilage degeneration, whereas SHP2 knockdown mitigated this adverse effect through increasing *Acan, Col2a1* and *Sox9* expression. Moreover, they found that SHP2 overexpression initiated cartilage catabolism by bolstering IL-1β-evoked β-catenin signaling, which then drove MMP3 and MMP13 production. Building on these findings, Tao et al. knocked down *Ptpn11* in murine AC via adenovirus-mediated shRNA expression. These mice, compared to controls, displayed attenuated OA severity after DMM surgery. However, some of Tao’s findings are confusing. First, whether SHP2 regulated IL-1β-evoked β-catenin signaling was not explicitly demonstrated as no direct evidence was provided that SHP2’s interaction with β-catenin in chondrocytes was IL-1β-dependent. Second, it is perplexing that *Ptpn11* knockdown decreased the total β-catenin level but did not impact β-catenin Y142 phosphorylation. Phosphorylation has long been considered as a key mechanism in regulating β-catenin stability and transcriptional activity (Fig. 6).^125^ β-catenin phosphorylation at Y142 serves as an index of β-catenin nuclear translocation and transcriptional activation,^126,127^ and β-catenin phosphorylation by GSK3 on S33/S37/T41 is crucial to induce its ubiquitination and subsequent proteasome degradation.^128,129^ In Tao’s work, IL-1β induced rapid dephosphorylation of β-catenin on S33/37/T41, which, however, had no impact on total β-catenin level. To follow up, we examined the effect of SHP2 ablation on IL-1β-evoked β-catenin signaling in murine *Prg4*^***+***^ cells. IL-1β hesitantly induced β-catenin phosphorylation (S33/S37/T41) and degradation, and either genetic or chemical ablation of SHP2 had little impact on this action. These findings were further confirmed by analogous studies with *Acan*^*+*^ murine chondrocytes.^93^ Based on these outcomes, we conclude that SHP2 deficiency has negligible, if any, influence on IL1-β-evoked β-catenin signaling in chondrocytes. The inconsistency between Tao’s and our findings warrants additional studies on SHP2’s regulation of β-catenin signaling in osteoarthritic cartilage.

### Does Shp2 deficiency promote IL-1β-evoked inflammation in osteoarthritic cartilage?

IL-1β signals through the IL-1R1, which recruits the co-receptor IL-1RAcP and forms a heterodimeric receptor complex, which in turn interacts with the intracellular adapter protein MYD88 and IL-1 receptor-associated protein kinases (IRAKs). IRAK activation and TRAF6 phosphorylation facilitate the association of TAK1 (mitogen-activated kinase kinase kinase 7, or MAP3K7) with TRAF6 and activation of MAPK pathways, including ERK, JNK and p38 MAPK, and TFs, such as NF-κB and AP-1 (activator protein 1).^130^ The activation of these pathways and TFs is involved in cellular responses to stress, cytokines, and inflammatory diseases (Fig. 6). Given that NF-κB controls many genes involved in inflammation, it is not surprising that NF-κB is chronically active in OA cartilage. Tao et al.^112^ described that SHP2 regulated IL-1β-evoked MAPK and NF-κB signaling in chondrocytes. SHP2 knockdown or overexpression either attenuated or enhanced, respectively, the activation of JNK, ERK1/2, and p38MAPK, suggesting that SHP2 is required for IL-1β-induced MAPK signaling. Aligning with these findings, increased or reduced levels of SHP2 promoted or hindered, respectively, the activation of NF-κB signaling in chondrocytes, manifesting an altered phosphorylation of IKKβ, p65RelA, and IkBα. Based on these findings, Tao and colleagues concluded that increased SHP2 expression augmented IL-1β-evoked inflammatory response by activating the MAPK and NF-κB signaling pathways and catabolic programs in cartilage. This statement, however, is largely based on signaling data gathered from a single time point. In some experiments, differences in the states or levels of signaling molecules existed prior to IL-1β stimulation, indicating that they were IL-1β-independent. Therefore, further examination of SHP2’s regulation of IL-1β-evoked MAPK and NF-κB signaling events is required to clarify these confusions. To look into whether *Ptpn11* knockdown ameliorated DMM-evoked OA in vivo, Tao and colleagues performed weekly intraarticular injections of *Ptpn11* shRNA-encoding adenovirus from 8 to 12 weeks of age. *Ptpn11* knockdown was found to delay cartilage destruction and osteophyte formation.^112^ However, the impact of such an aggressive intraarticular delivery protocol on synovium homeostasis and joint inflammation was neglected. We therefore examined the effect of SHP2 on IL-1β-evoked NF-κB signaling in murine chondrocytes. Although IL-1β rapidly induced IkBα phosphorylation and degradation, and p65RelA phosphorylation, SHP2 genetic deletion and chemical degradation had no apparent impact on the NF-κB signaling pathway.^93^ Clearly, the uncertainties in existing studies demand further inquiry into the association between SHP2 abundance and altered β-catenin signaling and inflammation in osteoarthritic AC.

## Do *Ptpn11*^*Ex4fx/fx*^*;Cd4*^*Cre*^ mice develop ankylosing spondylitis or cartilage tumor?

Recently, mice lacking SHP2 in the *Cd4-Cre* lineage were reported to develop cartilage tumors^95,96^ and ankylosing spondylitis-like disease.^104^ These findings were surprising at first glance considering the well-established expression of *Cd4-Cre* in the T cell lineage.^131,132^ However, they were actually well-grounded as the *Cd4* promoter was lately identified to be active in chondrocytes, and the skeletal diseases of *Cd4-Cre;Shp2*^*Ex11fx/fx*^ and *Cd4-Cre;Shp2*^*Ex4fx/fx*^ mice were shown to be T cell-non-autonomous.^96,104^ SHP2 has been speculated to regulate T-cell receptor (TCR) signaling, and the conditional inactivation of *Shp2* (*Shp2*^*Ex4fx/fx*^) in mice using *Lck-Cre* impaired T cell development.^133^ However, this finding was challenged by the work done by Miah et al. who studied the roles of SHP2 in lymphopoiesis and NK cell function. They indeed found that *Shp2* (*Shp2*^*Ex11fx/fx*^) could be robustly inactivated in the T cell compartment using *Lck-Cre* and that T cell development and function were normal in mutant mice.^95,96^ What caused the discrepancies between the two studies remains elusive. Nevertheless, *Cd4-Cre;Shp2*^*Ex11fx/fx*^ mice developed cartilage tumors upon aging^95,96^ that mimicked the clinical features of METCDS and the T cell-independent skeletal phenotypes of *Cd4-Cre;Sos1*^*fx/fx*^*, Cd4-Cre;Erk2*^*fx/fx*^*;Erk1*^*-/-*^, and *Cd4-Cre;Shp2*^*Ex4fx/fx*^ mice.^133–135^ Importantly, the neoplastic lesions of *Cd4-Cre;Shp2*^*Ex4fx/fx*^ and *Cd4-Cre;Shp2*^*Ex11fx/fx*^ mice comprised Cre-reporter-positive chondrocytes^96^ and could be ameliorated by deleting one *Sox9* allele.^96^ Congruently, these data indicated that the SOS/RAS/SHP2/ERK signaling axis is crucial to maintain cartilage homeostasis and that dysregulation of this pathway can cause excessive cell proliferation and cartilage tumor formation (Fig. 2b).

Interestingly, the skeletal phenotypes of *Cd4-Cre;Shp2Ex4*^*fx/fx*^ mice, e.g., kyphoscoliosis, ankylosis of the hip and knee, impeded fusion of epiphyseal GPs, and ectopic new bone formation, were interpreted as an ankylosing spondylitis (AS)-like disease by Shao et al. ^104^ Similar skeletal manifestations, however, were previously described in *Ctsk*^*Cre*^*;Shp2*^*Ex11fx/fx*^,^87,90^ *Col2a1-CreER;Shp2*^*Ex4fx/fx*^,^16,94,108^ *Agc1*^*CreER*^*;Shp2*^*Ex11fx/fx*^,^136^ *Bglap-CreER;Shp2*^*Ex11fx/fx*^,^137^ *Cd4-Cre;Shp2*^*Ex11fx/fx*^,^95,96^ *Cd4-Cre;Sos1/2*^*fx/fx*^,^134^ and *Cd4-Cre;Erk2*^*fx/fx*^*;Erk1*^*-/-*^ mice^135^ as the result of excessive cell proliferation in epiphyseal and vertebral cartilage, deformation of synovial joint and vertebra, and subsequent ossification. Importantly, the skeletal abnormalities of *Ctsk*^*Cre*^*;Shp2*^*Ex11fx/fx*^ mice could be ameliorated by blocking hedgehog signaling with the smoothened inhibitor (SMOi) PF-04449913,^90^ and this finding was recently confirmed by Shao et al. in *Cd4-Cre;SHP2*^*Ex4fx/fx*^ mice^104^ using a SMOi variant. Moreover, we carefully examined the *Ctsk*^*Cre*^*;Shp2*^*Ex11fl/fl*^ and *Agc1*^*CreER*^*;Shp2*^*Ex11fl/fl*^ mice and did not detect any sign of inflammation in the affected joints and neoplastic lesions. We conclude that SHP2 deletion in *Ctsk*^*+*^ and *Acan*^*+*^ chondroid cells does not elicit inflammation in cartilage and that it remains up for debate whether the skeletal disease observed in mice lacking SHP2 in *Cd4*^+^ cells is ankylosing spondylitis.

Shao et al.^104^ also reported that *Shp2* knockdown in chondrocytes in vitro via *shPtpn11* increased the abundance of BMP6 and led to BMP signaling-dependent SMAD1/5 phosphorylation in BMSCs and to ectopic bone formation. This statement was based on qRT-PCR data showing that the *Bmp6* transcript level increased about 2-fold in chondrocytes positive for *Ptpn11* shRNA. We carried out follow-up studies using RNAscope and found comparable levels of *Bmp6* transcript in hypertrophic chondrocytes of 4-week-old *Prg4*^*CreER*^_*;*_*Shp2*^*+/+*^ and *Agc1*^*CreER*^*;Shp2*^*Ex11fx/fx*^ mice compared to control mice, with all mice receiving 3 doses of tamoxifen at week 2. Further characterization of aged *Agc1*^*CreER*^*;Shp2*^*Ex11fx/fx*^ and *Ctsk*^*Cre*^*;Shp2*^*Ex11fx/fx*^ mice revealed that *Bmp6* transcripts were undetectable in GPC, AC, and cartilaginous lesions.^93^ We conclude that SHP2 deficiency in chondrocytes is unlikely to significantly impact *Bmp6* expression and its related BMP signaling and ossification. An additional study is required to warrant the importance of SHP2-modulated BMP6 signaling in ankylosing spondylitis.

While analyzing literature about the trajectory of METCDS, we noticed that it took much longer for cartilaginous lesions to form in *Bglap-CreER;Shp2*^*Ex11fx/fx*^*, Cd4-Cre;Shp2*^*Ex4fx/fx*^, *Cd4-Cre;Sos1*^*fx/fx*^ and *Cd4-Cre;Erk2*^*fx/fx*^*;Erk1*^*-/-*^ mice than in *Ctsk*^*Cre*^*;Shp2*^*Ex11fx/fx*^*; Col2a1-CreER;Shp2*^*Ex11fx/fx*^, and *Agc1*^*CreER*^*;Shp2*^*Ex11fx/fx*^ mice. These distinct time courses likely reflect, in part, the activity of the individual promoters driving Cre/CreER, the temporal requirement to develop LOH, and the slow clonal expansion of SHP2 mutant chondroid cells.

## Is SHP2 a pharmacological target for osteoarthritis?

Recent studies have revealed that SHP2 regulates the abundance of SOX9 and the expression of SOX9-dependent cartilage anabolic genes. This regulation occurs, at least in part, through PTMs of SOX9, which include phosphorylation and SUMOylation.^91^ It has been established that phosphorylation of SOX9 occurs at a serine (S) or threonine (T) residues in the AGC family kinase consensus site R/KxxS/T^138–140^ and SUMOylation occurs at a lysine (K) residue within the consensus motif ψKxD/E (where ψ denotes a large hydrophobic residue and x denotes any residue).^141,142^ Of note, SOX9 is also regulated by others PTMs (e.g., methylation and acetylation)^143^ but it is beyond the scope of this review to describe them. Importantly, SHP2 depletion leads to increased SOX9 SUMOylation and phosphorylation at the AGC kinase site haboring Ser181, and this phosphorylation can be interrupted by the PKA inhibitor KT5720, indicating that SHP2 influences SOX9 phosphorylation, at least through PKA^91^ (Fig. 6). These findings are further supported by the evidence that PKA agonists promote the expression of SOX9-responsive cartilage anabolic genes^144,145^ and provide a further premise for spatiotemporally targeting SHP2 signaling to improve chondrogenesis.

Small molecule protein kinase inhibitors have revolutionized cancer therapy in the past two decades,^146^ and evidence of their efficacy in tissue repair is emerging.^147,148^ The prochondrogenic effects of SHP2 deletion^16,90,91^ prompt further exploration of its translational applicability. Intra-articular injection of *Shp2* shRNA or SHP099, which suppresses SHP2 synthesis or allosterically inhibits SHP2 by stabilizing its auto-blocking conformation,^149^ was claimed to attenuate posttraumatic OA in mice^111,112^ and facilitate AC repair in a rabbit full-thickness cartilage defect model.^150^ It remains controversial, however, whether the outcomes resulted from reduced SHP2 expression or enzymatic activity. As stated earlier, SHP2 is ubiquitously expressed and supports the physiological functions of many tissues. Thus, systemic SHP2 inhibition for preventing or treating OA would be cautionally advised due to the chronic and long trajectory of this disease. Formulations that can be delivered intraarticularly and have a long half-life would be preferable. A drug that falls in this category is the SHP2 proteolysis-targeting chimera (PROTAC) SHP2D26,^151^ which was reported to be 30-times more potent than SHP099 and to have an extended duration of action.^149,151^ Articular chondrocytes treated with SHP2D26 for 12 h demonstrated robust SHP2 degradation (DC50 of ~25 nmol/L) and did not show signs of toxicity when exposed to 2 μmol/L of SHP2D26 for 48 h. Prolonged exposure of superficial (*Prg4*^***+***^) articular chondrocytes to SHP2D26 significantly increased SOX9 level and anabolic gene expression,^93^ suggesting that SHP2D26 has a promising translational potential.

## Summary and outlook

Ever since *PTPN11* variants were linked to METCDS, substantial research efforts have been devoted toward uncovering the ins and outs of its protein product, SHP2, in the chondrocyte lineage. To date, the roles of SHP2 in chondrocyte differentiation are fairly well understood, but more work is warranted to detail the whole spectrum of impact of SHP2 loss of function in cartilage. Less understood, despite a potential for breakthroughs, is the impact of SHP2 on chondroprogenitors. Key mechanisms involved in SHP2 regulation at the gene, epigenetic, RNA, and protein levels have been identified. However, this has highlighted that the SHP2 regulatory network is likely much more complex than currently recognized, and it plays a crucial role in ensuring proper development of growth plate chondrocytes, endochondral ossification, and longitudinal bone growth. Dysregulation of SHP2 has also been associated with cartilage degeneration and osteoarthritis. A important goal for future research is to further investigate the various mechanisms of SHP2’s action and regulation. Gaining definitive knowledge will involve employing cutting-edge approaches, both in vitro and in vivo, such as single-cell RNA sequencing (scRNAseq) and CRISPR technology, to identify and functionally test SHP2 targets, co-factors, and regulators. New findings are expected to provide valuable insights into the mechanisms underlying various cartilage diseases and to develop improved strategies for balancing chondrocyte function, extracellular matrix maintenance, and inflammation control, ultimately aiding in the treatment of these skeletal disorders.
